# Access to cellulose limits the efficiency of enzymatic hydrolysis: the role of amorphogenesis

**DOI:** 10.1186/1754-6834-3-4

**Published:** 2010-02-23

**Authors:** Valdeir Arantes, Jack N Saddler

**Affiliations:** 1 Forestry Products Biotechnology/Bioenergy Group, Department of Wood Science, Faculty of Forestry, University of British Columbia, 2424 Main Mall, Vancouver BC, V6T 1Z4, Canada

## Abstract

The efficient enzymatic saccharification of cellulose at low cellulase (protein) loadings continues to be a challenge for commercialization of a process for bioconversion of lignocellulose to ethanol. Currently, effective pretreatment followed by high enzyme loading is needed to overcome several substrate and enzyme factors that limit rapid and complete hydrolysis of the cellulosic fraction of biomass substrates. One of the major barriers faced by cellulase enzymes is their limited access to much of the cellulose that is buried within the highly ordered and tightly packed fibrillar architecture of the cellulose microfibrils. Rather than a sequential 'shaving' or 'planing' of the cellulose fibrils from the outside, it has been suggested that these inaccessible regions are disrupted or loosened by non-hydrolytic proteins, thereby increasing the cellulose surface area and making it more accessible to the cellulase enzyme complex. This initial stage in enzymatic saccharification of cellulose has been termed amorphogenesis. In this review, we describe the various amorphogenesis-inducing agents that have been suggested, and their possible role in enhancing the enzymatic hydrolysis of cellulose.

## Review

Continuing interest in the utilization of renewable biomass resources for the production of alternative fuels has brought increasing attention on the technical bottlenecks that still need to be resolved and how the variability of different lignocellulosic materials might influence the efficiency of enzymatic hydrolysis.

Over the past 40 to 50 years, many excellent research groups have been assessing the ability of carbohydrate-degrading enzymes to depolymerize the cellulosic component of lignocellulosic substrates into soluble, fermentable sugars. However the efficient, rapid and complete enzymatic hydrolysis of lignocellulosic materials using low protein loadings has proven to be one of the major technical and economical bottlenecks in the overall bioconversion process of lignocellulose to biofuels [1-4].

Several factors related to the substrates (such as lignin/hemicellulose association, degree of cellulose crystallinity and polymerization, extent of surface area) and enzymes (such as end-product inhibition, need for synergism, irreversible enzyme adsorption) have been suggested to account for the recalcitrance of cellulose to enzymatic hydrolysis [5]. However, there is still considerable disagreement in the literature regarding the relative importance of each of these factors, and our understanding of how enzymes completely hydrolyze cellulose is still far from complete.

Enzymatic saccharification of cellulose is generally described as a heterogeneous reaction system in which cellulases in an aqueous environment react with the insoluble, macroscopic and structured cellulose, containing highly ordered and less ordered regions. Unsatisfactorily, the majority of the research directed at understanding the mechanisms of cellulose biodegradation has given little attention to the existence and the influence that the fibrillar architecture of the cellulose fibril network will have on the enzyme reactivity and consequential course of heterogeneous cellulase reactions.

In order for cellulases to efficiently hydrolyze cellulosic substrates, they must first be able to access the cellulose chains that are tightly packed in the form of insoluble microfibrils encased in hemicellulose and lignin [5]. Previous work has shown that the ability of cellulase enzymes such as *Trichoderma reesei *cellobiohydrolase (CBH)I to access the cellulose chains within the microfibrils embedded in fiber walls is significantly limited, probably due to the enzyme's ability to access only the surface layers of the microfibrils [5]. Although cellulose could be slowly eroded by surface shaving or planing, it has been proposed that, to achieve efficient enzymatic saccharification, cellulose chains in the highly ordered and tightly packed regions of microfibrils should rather be delaminated, disrupted or loosened, thereby increasing the surface area and making the individual cellulose molecules more accessible and available for interactions with cellulose-degrading enzymes. Fiber swelling and fragmentation of cellulose aggregations into short fibers have been observed during enzymatic hydrolysis of cellulose before any detectable amount of reducing sugars is released [6-9]. This initial stage in enzymatic saccharification of cellulose has been termed amorphogenesis [6].

The original mechanistic model for enzymatic degradation of cellulose postulated by the pioneering work of Mandel and Reese introduced the C_1_-C_x _model [10,11]. They hypothesized that an unknown component of the cellulase system (C_1_, the so called 'swelling factor') opens up the cellulose matrix, allowing this now more accessible substrate to be depolymerized by the truly hydrolytic enzymes (C_x_) [10,11]. Although many hydrolytic enzymes that could account for the suggested C_x _action have been identified and characterized, so far the identification and characterization of the C_1 _factor remains elusive.

CBH 1, along with a number of other proteins (expansins, expansin-like proteins, swollenin), contains a polysaccharide binding surface. These proteins have been suggested to be able to non-hydrolytically loosen or disrupt the packaging of the cellulose fibril network. The cellulose-disrupting activity of such proteins has recently been shown to interact synergistically with cellulase enzymes when they are used to hydrolyze insoluble cellulose, apparently by increasing the accessibility of the cellulose to the enzymes [12,13]. In this review paper, we provide an overview of these amorphogenesis-inducing agents and their interactions with cellulose. In addition, their potential for possible application in the enzymatic saccharification of cellulose-containing materials for biofuel production is also discussed. The structural arrangement of the cellulose chains in the fibrillar architecture, and their accessibility and reactivity are also briefly outlined. The use of enzymes and their components is expected to radically influence the way we currently think cellulose is organized within the plant cell wall.

## Cellulose: structure, accessibility and reactivity

Cellulose, an insoluble polymer consisting of β-(1-4)-linked glucose residues [14-16], has been the subject of intense research for more than a century, and new insights into a better understanding of its molecular architecture continue to emerge [15,16]. It is well known that native cellulose molecules (cellulose I) are found in fibril form, and that its molecular architecture has a high degree of individuality, depending on its source (cell wall layer or plant type) [16].

Briefly, the visually dominant structural features of cellulose in higher plants are cellulose microfibrils with diameter of 2-10 nm, cross-linked by other cell wall components such as xyloglucans [15,16]. Microfibrils are unbranched fibrils composed of approximately 30-36 glucan chains aggregated laterally by means of hydrogen bonding and van der Waals forces to produce crystalline structures [15]. Microcrystalline cellulose has been shown to be made up of two different crystal phases: Iα and Iβ [15,16]. Although considerable progress has been made in elucidating the crystal structures of cellulose in microfibrils, they are still not well understood [15,16], and a deeper understanding of cellulose structure is required if we are to overcome the natural recalcitrance of lignocellulosic substrates. It is likely that these crystal structures affect the rate of diffusion of reactants and thus play an important role in the accessibility and reactivity of cellulose.

Previous work by Krässing [14] has shown that a higher degree of fibrillar aggregation produces a more compact fiber structure, with fewer, smaller interstices resulting in a smaller internal accessible surface area. An important feature of the highly ordered regions is that the cellulose chains are packed so tightly that even small molecules such as water cannot penetrate these highly organized structural entities [14]. The limited accessibility to these regions leads to alteration of their reactivity to swelling and reactive agents such as cellulases. With this type of structure, it is apparent that only the cellulose molecules situated on the surface of these aggregations would be susceptible to the degrading actions of enzymes.

If cellulose hydrolysis only occurs on the surface of the cellulose aggregations, the available surface area is a potential determinant of the maximum rate of hydrolysis that can be achieved. It has been proposed that the tightly packed cellulose regions are a major factor in contributing to the resistance of cellulose to degradation, by limiting the accessibility to cellulases [17,18]. In 1985, Coughlan [6] coined the term 'amorphogenesis' to suggest a possible mechanism by which the dispersion, swelling or delamination of cellulosic substrate occurred, resulting in a reduction in the degree of fibrillar aggregation and/or crystallinity, and the creation of a larger accessible surface by increasing the reactive internal surface. Consequently, amorphogenesis enhances the reactivity of the fibrous cellulosic substrates by increasing the amount of cellulose directly accessible to the enzymes.

**Figure 1:**
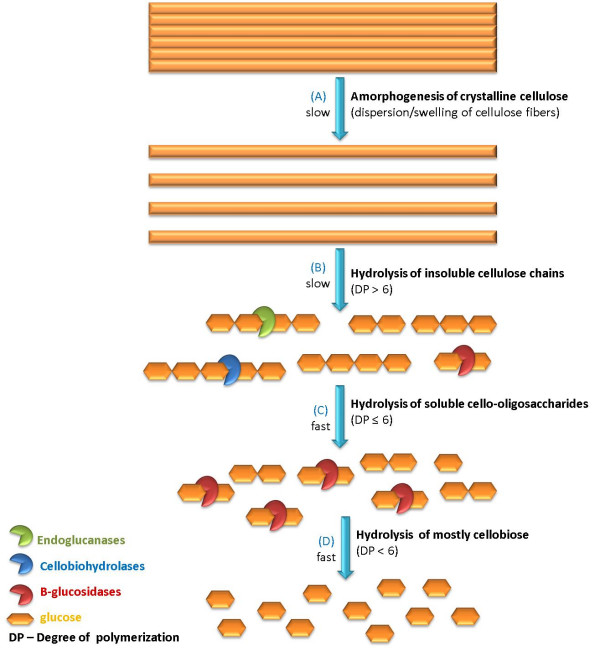
**Proposed mechanism for cellulose amorphogenesis/depolymerization by cellulases (adapted from **[6]**)**. Amorphogenesis (A) takes place at the macromolecular level by non-hydrolytic agents.

It has been suggested (Figure 1) that as cellulases need to adsorb onto the surface of the insoluble cellulose before hydrolysis, the inaccessible bulk of the substrate is structurally loosened to increase the molecular disorder of the tightly packed regions in the fibrous cellulosic network and to expose the cellulose chains buried within the microfibrils while they remain molecularly almost unchanged (amorphogenesis) (Figure 1a) [6]. Once the cellulose network is accessible to the enzymes, the synergistic action of endo- and exo-glucanases promote the fragmentation of accessible molecules to soluble cello-oligosaccharides (cellulosic molecules with a degree of polymerization of < 6 units) (Figure 1b), which are quickly hydrolyzed, mostly to cellobiose (Figure 1c). This component of the proposed mechanism seems likely to occur, as cello-oligosaccharides are seldom detected in solution, with cellobiose proving to be the primary cellulose hydrolysis product in most native cellulase systems. In most commercial cellulase systems, an extraneous source of β-glucosidase is usually added to completely hydrolyze the cellobiose to glucose (Figure 1d), enhancing the overall reaction by minimizing end-product inhibition.

## Carbohydrate-binding modules

Many carbohydrate-hydrolyzing enzymes, such as cellulases and xylanases, are modular proteins with at least two distinct modules: the catalytic module and the carbohydrate-binding module (CBM) [19]. CBMs are thought to have one or more of the following functions: enzyme concentration on the surface of the substrate/proximity effect (the phase transfer); substrate targeting/selectivity; and disruption of non-hydrolytic crystalline substrate. CBMs that are specific for insoluble cellulose can be grouped into two general categories: those that interact with crystalline cellulose (type A CBMs) and those that interact with non-crystalline cellulose (cello-oligosaccharides in addition to insoluble cellulose) (type B CBMs, [20] the so-called targeting function). These non-catalytic modules readily adsorb to accessible sites on a cellulose-containing substrate to form a complex held together by specific, non-covalent, thermodynamically favorable bonds [21]. Consequently, the catalytic module is aligned with the substrate to establish a high, local concentration of the enzyme on the cellulose surface (the so-called proximity function).

Various researchers have shown that removal of the CBM component of individual cellulases reduces the hydrolytic activity of the catalytic module on insoluble, crystalline substrates such as microcrystalline cellulose (Avicel), cotton, and filter paper, whereas their activity on soluble or amorphous cellulose remains largely unaffected [5,22,23]. In addition, CBMs isolated from both bacteria and fungi have been suggested to facilitate cellulose hydrolysis by physically disrupting the structure of the fibrous cellulosic network and releasing small particles, without showing any detectable hydrolytic activity, which is normally quantified by the release of reducing sugars (the so-called disruptive function) [7,8]. In recent studies investigating the morphological and structural changes of cotton fibers after treatment with purified CBM from fungal CBH1, it was found that CBM could promote non-hydrolytic disruption of crystalline cellulose by weakening and splitting the hydrogen bonds (as observed by infra-red spectroscopy and X-ray diffraction), thereby freeing cellulose chains [24,25]. Molecular dynamic simulations also provided a nanoscopic view of the mechanism, showing that strong and medium hydrogen bonds decreased dramatically when CBM was bound to the cellulose surface of cotton fibers [24]. Furthermore, CBM treatment of cellulosic fibers (Whatman CF11) has also been shown to reduce the interfiber interaction (disaggregation of agglomerates between the fibers, as observed by scanning electron microscopy) through steric and hydrophobic effects, which would increase the cellulose surface area [26].

Earlier workers [8] proposed that CBMs bind to the cellulose fibers and penetrate the fibrillar network at surface discontinuities, subsequently releasing cellulose fragments that are non-covalently associated with the fiber but bonded to the underlying microfibrils. They also suggested that further penetration by the CBM then exfoliates the fiber structure, releasing the ends of cellulose chains, which remain bound to the fiber, resulting in a roughening of the surface. In related work, Lee *et al. *[27], using atomic force microscopy, observed slightly elongated holes that were left throughout the surface of cotton fibers after they were treated with hexachloropalladate-inactivated *T. reesei *CBH I. It was suggested that these holes were a result of the penetration of the CBM into the cellulose fibers [27]. Incubation of cotton fibers with cellulase from *Thermotoga maritima*, which lacks CBM, had no effect on the surface of cotton fibers.

In a similar fashion to the proposed disruptive activity of CBM from CBH1, other CBMs have also been shown to display disruptive activity upon binding onto other non-soluble polysaccharides such as chitin (a cellulose derivative where the 2-hydroxy group is substituted with an acetamido group) and starch. One such component, CBP21 (~20 kDa), produced by *Serratia marcescens*, belongs to CBM family 33 and is known to bind to crystalline β-chitin (which has chitin chains arranged in a similar fashion to that of cellulose I) and strongly enhance chitin hydrolysis by chitinases [28,29]. This is thought to be due to increased substrate availability after disruption of the crystalline chitin structure [28,29]. It was suggested that the binding of CBP21 to chitin led to the disruption of the crystalline substrate structure through specific polar interactions that were not only important for binding, but also for alteration of the substrate structure [29]. In related work, Zeltins and Schrempf [28] showed that the chitin-binding protein CHB1 secreted by *Streptomyces olivaceoviridis *interacts specifically with crystalline α-chitin by binding and penetrating into the structure of the substrate. Another example of a CBM with disruptive activity comes from the starch-binding domain (SBD) of *Aspergillus niger *glucoamylase I, an exo-acting enzyme that releases glucose from the non-reducing ends of the polymer chains [30,31]. This glucoamylase contains an SBD with two binding sites for starch [30,31]. These sites have been shown to help with crystalline starch hydrolysis and also help promote disruption of α-glucan interchain binding at the surface of granular starch, thereby enhancing enzymatic degradation of crystalline starches by glucoamylase I [30,31].

**Figure 2:**
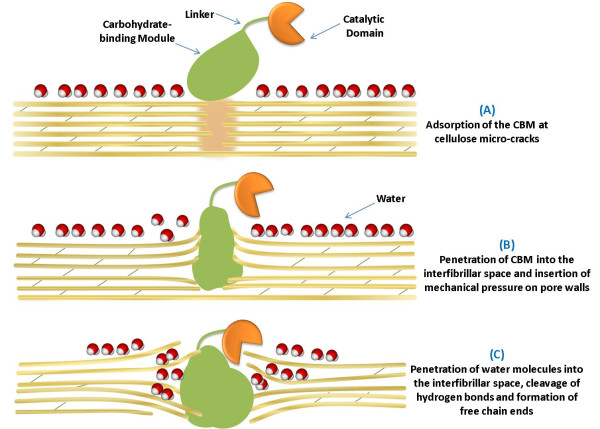
**Schematic representation of amorphogenesis of cellulose fibers mediated by the carbohydrate-binding module (CBM) of cellobiohydrolase I (CBHI) (adapted from **[36]**)**. For clarity, the carbohydrate-binding module is oversized compared with the catalytic domain.

Building on the C_1_-C_X _model of cellulose hydrolysis, Russian researchers [32-35] proposed a mechanism to try to explain the dispersion of cellulose [30-33] (Figure 2). They proposed that cellulases are adsorbed to cellulose defects (disturbances in the crystalline structure of cellulose, such as microcracks) (Figure 2a), followed by their penetration into the interfibrillar spaces (Figure 2b). This consequently would induce a mechanical action (dispersion) of the cellulose structure. It was suggested that the presence of the large enzyme within such a narrow space causes an increase in the mechanical pressure exerted on the cavity walls, swelling the cellulose structure and accommodating more and more water molecules between the microfibrils (Figure 2c). The water within the defects penetrates further and further inside the capillary space, breaking the hydrogen bonds between the cellulose chains, resulting in the disassociation of the individual microfibrils (Figure 2c). In turn, the adsorbed enzymes prevent the solvated chains and free chain ends from realigning and readhering [32,36].

Recent computational simulations have indicated that the water solutions in contact with microcrystalline cellulose surfaces are highly structured and that these structured water layers might inhibit molecular diffusion close to the cellulose surface [37]. During enzymatic hydrolysis, this would limit the approach of cellulases towards the cellulose surfaces [37]. More recent computer simulation studies with *T. reesei *CBH I action on microcrystalline cellulose Iβ showed that the CBM derived from this enzyme showed no tendency to dissociate from the cellulose surface [38], although it was observed to move about slightly on the surface [39]. This finding indicated that the suggested structured water layers may not be as problematic as originally suggested. Once the enzyme is adsorbed onto the cellulose surface, a processive hydrolysis mechanism would be faster than a mechanism that requires diffusion away from and subsequent repenetration of the hydration layers [37].

Although the functions of CBMs during enzymatic hydrolysis of cellulose have not been fully elucidated and continue to be the subject of research, it seems reasonable to believe that the primary role of a CBM is to anchor the catalytic module to cellulose. This anchoring by the CBM is generally accepted to increase the effective concentration of cellulases onto the solid substrate, thereby assisting the enzyme through the phase transfer from the soluble fraction (enzyme) to the insoluble fraction (substrate) [40]. Complementing this, CBM may also have a more active role in the depolymerization of cellulose by influencing the cellulose structure through the non-hydrolytic release of single cellulose chains from the highly ordered and tightly packed regions of microfibrils, which might occur by disrupting the intermicrofibrillar associative forces [6,32-36] and by feeding the newly exposed chains through the tunnel-shaped catalytic module for hydrolysis.

## Expansins

Expansins are plant-derived proteins, which were first identified in the early 1990s and are primarily known for their unique 'loosening' effect on the cellulosic network within plant cell walls during growth [41-43].

Two families of expansins have currently been characterized: α- and β-expansins [44,45]. Although they share only about 20% of their amino acid identity, they are of similar size (~27 kDa), contain a number of conserved residues and characteristic motifs distributed throughout the length of the protein, and their predicted secondary structures share up to 75% identity. However, they appear to act on different cell wall components [44,45].

Expansins usually consist of two domains (D1 and D2) connected by a peptide linker [44,45]. D1 shows structural and sequence similarity to the catalytic site found in family-45 glycosyl hydrolyses (GH45) whose members have been characterized as endoglucanases [44-48]. Although D1 has conserved much of the GH45 catalytic site, it lacks hydrolytic activity on cell wall polysaccharides [42,43]. Recently, Yennwar *et al. *[44] suggested that expansins do not display hydrolytic activity due to lack of a second aspartate (a key part of the catalytic machinery required for glucan hydrolysis by GH45 enzymes) in D1 [44]. D2 was initially speculated to be a CBM on the basis of conserved aromatic and polar residues on the surface of the protein [44,46]. However, recent studies on the structure of expansins have identified two potential polysaccharide binding surfaces, one of which corresponds to the buried D2 face contacting D1 [44]. In addition, the findings that the linker coupling D1 and D2 is very short and that the multiple contacts between D1 and D2 could enable close coupling of the two domains suggest that the two domains, when closely packed and aligned together, could form a potential polysaccharide-binding surface spanning D1 and D2 [44].

Most evidence suggests a non-hydrolytic action of expansins that enlarges cell wall cavities by binding polysaccharides and disrupting non-covalent bonds within cellulose microfibrils and between other cell wall polysaccharides attached to the microfibrils [44,45]. In addition, Yennawar *et al. *[44] suggested that expansins act as a molecular device that uses the strain energy stored in a taut cellulose-binding glycan to help dissociate the glycan from the surface of cellulose.

Despite the lack of hydrolytic activity in expansins themselves, some studies have shown that expansins enhance the enzymatic hydrolysis of crystalline cellulose by cellulases [49]. It has been proposed that this synergistic action is a result of the expansins making the glucan chains within the microfibrils more accessible to the cellulases [45]. In this model, expansins are believed to act like a zipper opening the crosslinking of cellulose microfibrils by ungluing the chains that stick them together, which in turn enhances cellulose accessibility, thereby speeding cellulase action [50,51]. For instance, Baker *et al. *[10], using yellow poplar sawdust pretreated with dilute acid, showed that extremely small additions of expansin along with *T. reesei *cellulases (ratio ~0.012) was sufficient to induce up to a 13% increase in cellulose conversion compared with the sugar yield obtained when cellulase was used alone.

## Expansin-like proteins

Some proteins produced by bacteria and fungi have been shown to have sequence similarity to plant expansins [10,11,52-56]. Kerff *et al. *[52] determined the structure and activities of one of the proteins secreted by *Bacillus subtilis*, a Gram-positive soil bacterium capable of colonizing the surface of plant roots. These authors considered this protein (EXLX1) to be a member of the expansin superfamily [52], based on its structural similarity to plant expansins (including its two-domain structure, with the precise spatial alignment of the two domains resulting in an open binding surface spanning both domains), its ability to bind to cell walls, its plant cell wall extension activity and its lack of hydrolytic activity against major polysaccharides of the plant cell wall.

When EXLX1 was used along with low levels of *T. reesei *cellulase enzymes (ratio 10:1) to hydrolyze microcrystalline cellulose (Avicel), it enhanced cellulose hydrolysis but not beyond the enhancement observed with bovine serum albumin (BSA), which was used as a nonspecific control [52]. This lower cellulolytic enhancing activity was attributed to the weak plant cell wall extension activity of EXLX1 (10-fold weaker than that of plant β-expansins) [52]. Under such hydrolysis conditions (high EXLX1:cellulase enzymes ratio), it cannot be ruled out that the higher concentration of EXLX1 in comparison to cellulase enzymes might have resulted in competition for binding sites between cellulases and EXLX1. Because BSA only loosely binds to Avicel [57], competition for binding sites between cellulases and BSA is not expected. This would explain why the cellulolytic enhancing activity of EXLX1 was lower than that of BSA. In contrast to the cellulolytic enhancing activity observed with EXLX1, the enhancement obtained by the addition of BSA was not a result of disrupting activity. It has previously been shown that cellulase enzymes adsorb to the inner wall of the reaction vessel during hydrolysis [58,59]. Thus, when high concentrations of BSA relative to cellulase enzymes (10:1) were used, it is likely that this high protein addition prevented or at least reduced adsorption of cellulases to the wall of the reaction vessel, resulting in more enzymes being available to react with the Avicel. This would lead to higher cellulose conversion compared with hydrolysis being carried out in the absence of BSA.

In related work, the EXLX1 gene was expressed in *Escherichia coli*; the purified recombinant protein displayed cellulose-binding and cellulose-weakening activities towards filter paper, indicating its functional homology with plant expansins [11]. Moreover, at much lower EXLX1:cellulase enzymes ratios than those used in previous work [52], the recombinant EXLX1 protein was found to promote significant cellulolytic enhancing activity when mixed with a commercial *T. reesei *cellulase mixture during hydrolysis of filter paper. This was shown when it was compared with the control containing only filter paper and cellulase and with the negative control containing filter paper, BSA and cellulase enzymes [11]. The ratio of the recombinant EXLX1 protein and cellulase enzymes was found to be a crucial determinant of the cellulolytic enhancing activity, with the highest synergistic activity (5.9-fold) observed at the lowest cellulase loading (0.012 filter paper units (FPU)/g filter paper) and the highest recombinant protein loading (300 μg/g filter paper). However, under this low cellulase loading, the cellulose conversion was < 10% of the theoretical maximum, and at higher cellulase loading (0.6 FPU/g filter paper, giving ~20% cellulose conversion), the synergistic activity was insignificant [11].

Another example of expansin-like proteins is a protein isolated from *T. reesei*, a well-known cellulolytic fungus [53]. This expansin-like protein (named swollenin due to its ability to swell cotton fibers) contains an amino-terminal fungal-type cellulose-binding module linked to the plant expansin homologous module [53]. Saloheimo *et al. *[53] reported the sequence similarity of swollenin to the fibronectin (Fn)III-type repeats of mammalian titin proteins. These latter proteins have been shown to be able to unfold and refold easily, allowing the protein to stretch. This ability might be important for swollenin if its function is to allow slippage of cellulose microfibrils in plant cell walls, as suggested for expansins.

Swollenin has also been shown to disrupt the structure of the cotton fibers, weaken filter paper and promote an apparent dispersion of *Valonia *cell wall structure [53]. This ability to disrupt solid substrates is unlikely to be the result of hydrolytic activity, as no reducing sugars were detected [53]. This would seem to indicate that swollenin is inactive against the β-1,4-glycosidic bonds in cellulose, suggesting that swollenin may share a similar role with expansins in swelling the cellulosic network within cell walls. Saloheimo *et al. *[53] have reported that swollenin is an important component in the enzyme mixture required for degradation of lignocellulosic biomass and hence, a potential candidate for the C_1_-induced dispersion proposed by Reese *et al. *[10]. In addition, there is evidence [53] that the swollenin gene is regulated in a manner similar to that of the *T. reesei *cellulase genes, so that low expression levels occur in the presence of glucose and high expression levels occur in the presence of cellulose [53].

The observation that microbial proteins containing an expansin-like domain, such as swollenin in *T. reesei *[53] and EXLX1 protein in *B. subtilis *[52], can enhance root colonization, suggest that expansin-type modules have been adapted by diverse microbes to facilitate their interactions with plants [52]. It seems that several swollenin-like activities are displayed by *T. reesei*, which may vary in their modes of action but would contribute synergistically to the efficient hydrolysis of the plant polysaccharides [53]. Similarly to the potential role of expansins in enhancing the efficiency of enzymatic hydrolysis of cellulose, it has been suggested that swollenin would increase the access of cellulases to cellulose chains by promoting dispersion of cellulose aggregations, exposing individual cellulose chains to interactions with cellulases. Although the cellulolytic enhancing activity of swollenin has not been assessed, recently a chimeric enzyme associating *T. reesei *swollenin with an *Aspergillus niger *feruloyl esterase was constructed and found to significantly increase the efficiency of ferulic acid release from lignocellulosic substrate [60].

## Yellow affinity substance

It has been shown that some cellulolytic bacteria, especially strains of the thermophilic anaerobic *Clostridium thermocellum*, produce an unidentified, yellow, water-insoluble substance when growing on cellulose [61,62]. Similarly to CBMs in fungal cellulases, this yellow substance has been shown to have a strong affinity for crystalline cellulose and to be part of the bacterial cellulolytic system required for efficient enzymatic degradation of cellulose [61-63]. Production of this 'yellow affinity substance' has been observed to precede the production of cellulases and also to be involved in the hydrolysis of cellulose by facilitating the binding of the cellulolytic enzyme complexes to cellulose [61,64]. Kopecny *et al. *[62] showed that endoglucanase and cellobiohydrolase activities were increased in the presence of the yellow affinity substance.

Despite some similarities in functions to that of CBMs, no substantial research has subsequently been conducted to investigate the exact means by which the yellow substance enhances cellulose saccharification.

## Other non-hydrolytic proteins

Recently, an unknown non-hydrolytic protein (Zea h), of approximately 56 kDa, purified from fresh postharvest corn stover (the unused plant parts left after harvest), was shown to decrease the hydrogen-bond intensity and crystallinity index of filter paper [65]. It also increased the adsorption of cellulase onto cellulosic substrates, which in turn increased the conversion of cellulose to glucose by a factor of 3.2, and accelerated hydrolysis by increasing hydrolysis rate of cellulases by a factor of 2 [65]. Although the Zea h protein appears to have potential to enhance the cellulolytic activity of cellulase enzymes, the mechanism involved in this enhancement and the three-dimensional structure of the protein remain to be resolved.

Several fungal proteins with homology to family 61 glycosyl hydrolase (GH61) have also been reported to show cellulolytic enhancing activity on a variety of pretreated lignocellulosic substrates when combined with *T. reesei *cellulases [66,67]. For instance, the expression of *Thielavia terrestris *GH61 in *T. reesei *allowed for a reduction in protein loading of 1.4-fold to reach 90% conversion of the cellulose in corn stover pretreated with steam [67]. Based on the lack of hydrolytic activity of GH61 on pretreated lignocellulosic substrates and on a variety of cellulosic and hemicellulosic model substrates, it was suggested that the cellulase-enhancing effect of such proteins is limited to substrates containing other cell wall-derived materials such as hemicellulose or lignin [67]. However, no clear correlation was observed between the proportion of these non-cellulolytic components and the degree of enhancement observed [67]. Although it has not been experimentally established, rather than acting on cellulose microfibrils themselves, GH61 proteins could be acting via disruption of non-covalent bonds between cellulose and the non-cellulolytic materials (as observed with some expansins [68]), resulting in increased access of cellulases to the cellulose microfibrils and enhancing the overall cellulolytic activity of the cellulase complex.

*T. reesei *Cel61B, which was previously thought to be an endoglucanase [69], is the only GH61 protein so far to have its three-dimensional structure resolved [70]. The structure appears to lack any suitable catalytic centre. However, a possible catalytic role has been speculated for the bound cation (nickel or other transition metals), given the highly conserved binding site in the GH61 proteins [70]. CBP21, a non-catalytic carbohydrate binding protein reported to disrupt the insoluble crystalline β-chitin structure and enhance chitin hydrolysis by chitinases as described earlier, is the protein whose structure is most similar to that of Cel61B. It is possible therefore that Cel61B may also have some direct or indirect role in the enzymatic degradation of cellulose [70]. However, the exact mechanism and function of Cel61B and other related GH61 proteins has yet to be fully resolved.

Low molecular weight peptides or phenolate-type compounds produced by 'brown rot' wood-decaying fungi (mainly Basidiomycota) are thought to mediate the non-hydrolytic/nonenzymatic attack of the lignocellulose matrix [71-73]. This attack is thought to increase pore size, consequently enhancing the diffusion of cellulases within the substrate [71-78]. These nonhydrolytic/nonenzymatic reactions mediated by low molecular weight compounds have been shown to enhance the activity of commercial cellulases and brown rot endoglucanases during hydrolysis of pure cellulose and various lignocellulosic substrates [79,80]. In addition, it has been suggested that this initial attack swells the ordered packing of the cellulose chains, exposing new end-groups of the fibrous cellulosic substrate (enhancing accessibility) to the attack of cellulases, as evidenced by a significant decrease in the crystallinity of the cotton fibers [81]. When the overall modification of milled spruce wood was examined using pyrolysis-molecular beam mass spectrometry coupled with multivariate analyses, it was apparent that the non-hydrolytic/nonenzymatic-mediated reactions could more readily open the structure of the lignocellulosic matrix, freeing cellulose fibrils [78], which indicated that this non-hydrolytic/nonenzymatic mechanism could be, in brown rot fungi, a potential candidate for the C_1_-induced disruption proposed by Reese *et al. *[10].

## Conclusion

Considerable progress has been made in elucidating the nature, type and mechanism of cellulases when soluble, short chain oligosaccharides are assumed to be the substrate. However, when the recalcitrant, largely inaccessible nature of the cellulosic substrate is considered, the exact biochemical mechanisms involved in the delamination, dispersion and swelling of cellulose has been much discussed but still remains largely unknown. It has been suggested that disruption of the highly ordered and tightly packed regions of the cellulose structure facilitates the exposure of inaccessible cellulose chains buried within these regions, thereby enhancing enzyme access to cellulose, which is expected to speed the hydrolytic attack of cellulases. In this context, some proteins have been proposed as having an active role in the solubilization of cellulose by affecting (weakening, swelling) the cellulose structure via the non-hydrolytic release of the previously enzyme-inaccessible individual cellulose chains. Although the mechanism by which each of these proteins attack cellulose has yet to be resolved, the observation that most of these swelling or delaminating agents contain a (potential) carbohydrate-binding surface may indicate that this binding module may play an important role in this non-hydrolytic amorphogenesis activity. It is apparent that further research is needed to better understand the possible mechanisms of these proposed amorphogenesis-inducing agents. Moreover, it is also possible that the enzymatic hydrolysis of cellulose occurs as just an external surface phenomenon. However this is unlikely as, although relatively slow, the rate of cellulose hydrolysis indicates that there must be some creation of new surfaces within the cellulose matrix. However, how this delamination, swelling and dispersion action of the cellulase complex occurs has yet to be fully determined.

## Competing interests

The authors declare that they have no competing interests.

## Authors' contributions

VA and JNS conceptualized, researched and wrote the manuscript. All authors read and approved the final manuscript.
